# Clinical characteristics and prognosis of primary leiomyosarcoma of the pancreas: a systematic review

**DOI:** 10.1186/1477-7819-11-290

**Published:** 2013-11-12

**Authors:** Jianwei Xu, Taiping Zhang, Tianxiao Wang, Lei You, Yupei Zhao

**Affiliations:** 1 Department of General Surgery, Peking Union Medical College Hospital, Chinese Academy of Medical Sciences and Peking Union Medical College, No1 Shuaifuyuan, Wangfujing Street, Beijing 100730, China

**Keywords:** Pancreatic tumor, Leiomyosarcoma, Sarcoma, Resection, Prognosis

## Abstract

**Background:**

Primary pancreatic leiomyosarcoma (PLMS) is rare. The clinical characteristics and prognosis is still not completely understood. The aim of the present study is to identify the clinical characteristics and long-term outcomes of PLMS from the existing reported cases in different scientific literature.

**Methods:**

PLMS cases reported in Chinese and English journals were collected and reviewed. Clinical features and long-term outcomes of these cases were summarized and analyzed statistically.

**Results:**

A total of 69 cases reported from both Chinese and English journals were included in the present study. An equal incidence in gender was observed. The mean age was 53.9 ± 14.7 years. The most common symptoms were abdominal mass, abdominal pain, and weight loss. The mean size of the tumor was 11.4 ± 7.1 cm. The incidence of PLMS between the head and body-tail of the pancreas had a similar pattern. Twenty-five percent of patients had distant metastasis and 19% of patients had adjacent organs/vessels invasion at the time of diagnosis. But lymph node metastasis was documented in only one (1.5%) patient. The median survival time was 48 months. The overall 1-, 3-, 5-, and 10-year survival rates were 66.6%, 51.2%, 43.9%, and 29.3%, respectively. Results from the multivariate analysis showed that non-radical resection (*P* = 0.000; hazard ratio (HR) 5.128; 95% confidence interval (CI) 2.041-12.987) was the independent adverse prognostic factor. Adjacent organs/vessels invasion (yes) may be considered as an another potential independent adverse prognostic factor (*P* = 0.071; HR 2.708; 95% CI 0.981-7.474).

**Conclusions:**

PLMS is rare without specific clinical features. PLMS is an aggressive tumor and has a poor prognosis. Radical resection can prolong survival time of the patients.

## Background

Primary leiomyosarcoma is an extremely rare mesenchymal tumor of the pancreas, which accounts for 0.1% of malignant pancreatic tumors [1]. Primary pancreatic leiomyosarcoma (PLMS) was first described by Ross in 1951 [2], and to date 64 cases have been reported [3]. However, most of them have been documented as single cases, with clinical characteristics and prognosis which cannot be completely understood. To understand the characteristics and long-term outcomes of PLMS, the present study reviews earlier reported cases in Chinese and English journals across the globe.

## Methods

The systematic literature search was performed via China Knowledge Resource Integrated (CNKI) database to identify the cases reported in Chinese journals, with the last search on 1 March 2013. A similar search was made in MEDLINE, EMBASE, and the Cochrane databases for cases reported in English journals. The primary key words used were ‘pancreas and leiomyosarcoma’, ‘pancreatic and leiomyosarcoma’, ‘pancreas and mesenchymal tumor’, ‘pancreatic and mesenchymal tumor’, ‘pancreas and sarcoma’, and ‘pancreatic and sarcoma’. Only the cases originated from the pancreas were included in the study. Clinical features and long-term outcomes of these cases were summarized and analyzed.

SPSS v.13.0 (SPSS, Inc., Chicago, IL, USA) was used for all analyses. The Pearson *χ*^2^ test and Fisher’s exact test were used to compare categorical variables. The Kaplan-Meier method was used to calculated overall survival (OS) using the log-rank test. Cox regression analysis was performed for multivariate survival analysis. A two-sided *P* value <0.05 was considered statistically significant.

## Results

### Demographic and clinical data

Twenty cases were reported in Chinese journals (Additional file 1), and 49 cases were reported in English journals [1,2,4-35] (Additional file 2). A total of 69 cases were included in the present study. The gender of 68 patients in these 69 cases was documented, which was 35 men and 33 women. An equal incidence in gender was observed (male-female ratio of 1.06) in the data. The mean age was 53.9 ± 14.7 years (median, 53 years; range, 14-87 years).

Symptoms were described in 63 cases and of which 58 patients had symptoms. The most common symptoms were abdominal mass (29 cases), abdominal pain (25 cases), and weight loss (19 cases). Other symptoms like jaundice (8 cases), anemia (6 cases), gastrointestinal bleeding (5 cases), and vomiting (5 cases) were also reported.

The tumor location was documented in 63 cases. The tumors were located in the head of the pancreas in 30 cases, and in the body-tail in 32 cases. One case had tumor in the whole pancreas. The mean size was 11.4 ± 7.1 cm (median, 10 cm; range, 1-30 cm). The gross morphology was recorded in 49 cases. Twenty-six tumors were solid mass, eight tumors were cystic mass, and fifteen were mixed mass.

The status of metastasis and regional invasion at the time of diagnosis were identified in 68 patients. Seventeen (25%) patients had distant metastasis and 13 (19%) patients had adjacent organs/vessels invasion. Lymph node metastasis was documented in only one (1.5%) patient. Men had a higher incidence of metastasis and regional invasion than women (19/35 *vs.* 11/33; *P* = 0.082). The surgical treatment method was illustrated in 62 cases, of which 40 cases achieved radical resection, and 22 cases underwent palliative resection or biopsy.

### Survival analysis

Forty-nine cases with long-term outcomes data were included in the survival analysis. The median survival time was 48 months. The overall 1-, 3-, 5-, and 10-year survival rates were 66.6%, 51.2%, 43.9%, and 29.3%, respectively. Univariate analysis showed that age ≥55 years, distant metastasis (yes), adjacent organs/vessels invasion (yes), and radical resection (no) were the potential adverse prognostic factors (Table 1). Multivariate analysis of the cases showed that non-radical resection (*P* = 0.000; hazard ratio (HR) 5.128; 95% confidence interval (CI) 2.041-12.987) was the independent adverse prognostic factor. Adjacent organs/vessels invasion (yes) might be also considered as another independent adverse prognostic factor (*P* = 0.071; HR 2.708; 95% CI 0.981-7.474) (Table 2).

**Table 1:** Univariate analysis of potential prognostic factors

**Variables**	**Survival time (median ± SE, year)**	**1-year survival rate, %**	**3-year survival rate, %**	***P *** **value**
Gender				0.398
Male	33 ± 15.8	58.4	41.7	
Female	98 ± 55.5	73.2	57.6	
Age (years)				0.049
< 55	None	72.7	68.1	
≥ 55	22 ± 7.9	59.1	27.9	
Locations				0.477
Head	48 ± 26.5	70.8	58.8	
Body-tail	33 ± 16.4	71.2	41.9	
Size (cm)				0.092
< 10	33 ± 17.6	74.3	67.5	
≥ 10	None	67.9	43.6	
Gross morphology				0.142
Solid	None	75.6	56.7	
Cystic	12 ± 3.3	40	20	
Mixed	None	80	80	
Distant metastasis				0.007
Yes	12 ± 6.3	50.0	0	
No	98 ± 46.1	76.6	63.6	
Adjacent organs/vessels invasion				0.011
Yes	6 ± 1.4	25.0	25.0	
No	48 ± 29.4	82.8	56.6	
Radical resection				0.000
Yes	98 ± 45.2	80.5	72.2	
No	8 ± 3.5	40.3	0	

Due to censored data ≥50%, the median survival time cannot be calculated.

A two-sided *P* value <0.05 was considered statistically significant.

SE, standard error.

**Table 2:** Multivariate analysis of potential prognostic factors

**Variables**	***P *** **value**	**Hazard ratio**	**95% Confidence interval**	
			**Lower**	**Upper**
Adjacent organs/vessels invasion (yes)	0.071	2.708	0.981	7.474
Radical resection (no)	0.000	5.128	2.041	12.987

A two-sided *P* value <0.05 was considered statistically significant.

## Discussion

PLMS is rare. Earlier studies by Baylor et al. showed only five leiomyosarcomas among 5,057 pancreatic malignant tumors screened [1]. PLMS is considered to originate either from the smooth muscle region of the pancreatic ducts or the wall of small intra-pancreatic vessels [4]. Leiomyosarcomas originating from the stomach, duodenum, and retroperitoneal organs often invade the pancreas, mimicking PLMS [36]. A definite diagnosis of PLMS is needed to rule out tumors originating from these adjacent organs.

A generally equal incidence of PLMS in gender was observed. There were 35 men and 33 women with a ratio of 1.06. The age of the patients ranged from 14 to 87 years (median, 53 years). The most common symptoms observed in the patients were abdominal mass, abdominal pain, and weight loss, but no specific symptoms were recorded from any of the patients diagnosed with PLMS. Small tumors usually had no symptoms and were found incidentally [36]. The mean size of tumors was 11.4 ± 7.1 cm (range, 1-30 cm). Twenty-three of 49 tumors were mixed or cystic mass. Large tumors with cystic degeneration presented a highly aggressive malignancy, such as adjacent organ invasion and distant metastasis [6]. The tumors were located in the head of the pancreas in 30 cases and in the body-tail in 32 cases. There was a similar incidence between the head and body-tail.

Due to its presence as an obvious mass, the tumor can be found easily by imaging studies. Trans-abdominal ultrasonography (TAUS), computed tomography (CT), positron emission tomography and computed tomography (PET/CT), magnetic resonance imaging (MRI), angiography, and endoscopic ultrasonography (EUS) were all effective in diagnosing the PLMS, but there are no specific imaging features [7]. PLMS appears as a heterogeneous, hypervascular mass on CT scan. As the tumor volume increases, hemorrhagic, necrotic, and cystic changes can be observed [5,8]. Tumors with cystic lesions can be confused with pseudocysts of the pancreas [6,9]. PLMS appears as a high incidence of distant metastasis and regional invasion with rare lymphatic involvement, which could be the key point to differential diagnosis. It is difficult to do preoperative qualitative diagnosis by non-invasive diagnostic method. CT or US guided fine-needle aspiration biopsy (FNAB) is an alternative diagnostic method for PLMS. Three of four cases showed a definite diagnosis through US-guided FNAB reported in literature [3].

The accurate diagnosis of PLMS depends mainly on the histological examinations and immunohistochemical staining. Histologically, PLMS showed well-formed fascicles of spindle cells with blunt-ended nuclei intersecting at vertical angles, varying degrees of pleomorphism, and a substantial number of mitosis [10,11]. However, it is difficult to histologically differentiate PLMS from inflammatory myofibroblastic tumors and non-myogenic spindle cell sarcomas, namely fibrosarcomas, malignant fibrous histiocytomas, and malignant peripheral nerve sheath tumors [10]. Therefore, an immunohistochemical staining is crucial for an accurate diagnosis. Immunohistochemical examinations have an important value in evaluating spindle cell tumors which were supposed to be of smooth muscle origin. These tumors are positive for muscle markers (such as α-smooth muscle actin, MSA, and desmin), with negative expression of epithelial (such as cytokeratin, EMA, and CEA) and neural (such as S100 protein) markers [10].

PLMS is an aggressive tumor. In the present systematic review, 25% of patients had distant metastasis and 19% of patients had adjacent organs/vessels invasion at the time of diagnosis. Lymph node metastasis was not common which was documented only in one patient (1.5%). This feature helps us to differentiate PLMS from other pancreatic malignant tumors and more surgeons prefer to perform a local radical resection rather than extensive node dissection based on this feature. Gender may be related to the degree of malignancy. Men seemingly had a higher incidence of metastasis and regional invasion than women (*P* = 0.082).

Survival analysis showed that median survival time of PLMS was 48 months. The overall 1-, 3-, 5-, and 10-year survival rates were 66.6%, 51.2%, 43.9%, and 29.3%, respectively. From univariate analysis, we showed that age ≥55 years, distant metastasis (yes), adjacent organs/vessels invasion (yes), and radical resection (no) were the potential adverse prognostic factors of PLMS. Multivariate analysis indicated that non-radical resection was the independent adverse prognostic factor. Radical resection was associated with prolonged overall survival time. Adjacent organs/vessels invasion (yes) might be another independent adverse prognostic factor, but statistical difference was not observed (*P* = 0.071). Although the dimension of tumors is an important indicator with regard to resectability, large tumor size was not an effective predictor of adverse prognosis in the current study. Mitotic counts >10 mitoses per 10 high-powered fields (HPFs) was reported as another adverse predictor [10]. Since data of this retrospective study extracted from the literature were not in detail, a comprehensive prognosis analysis could not be performed.

## Conclusion

PLMS is a very rare malignant tumor of the pancreas. Clinical features and imaging examinations are no specific. The accurate diagnosis of PLMS depends on histological examinations and immunohistochemical staining. PLMS is an aggressive tumor with poor prognosis. Radical resection can prolong survival time of the patient.

## Abbreviations

CEA: Carcinoembryonic antigen; CI: Confidence interval; CNKI: China Knowledge Resource Integrated database; CT: Computed tomography; EMA: Epithelial membrane protein; EUS: Endoscopic ultrasonography; FNAB: Fine-needle aspiration biopsy; HPFs: High-powered fields; HR: Hazard ratio; MSA: Muscle-specific actin; MRI: Magnetic resonance imaging; OS: Overall survival; PET/CT: Positron emission tomography and computed tomography; PLMS: Primary pancreatic leiomyosarcoma; TAUS: Trans-abdominal ultrasonography.

## Competing interests

All the authors declare no competing interests.

## Authors’ contributions

Xu drafted the manuscript. Zhang and Zhao designed the study and helped in drafting the manuscript. Xu, Wang, and You collected the data and performed the statistical analysis. All authors have read and approved the final manuscript.

## Supplementary Material

Additional file 1 Reported cases of PLMS in Chinese literature. Click here for file

Additional file 2 Reported cases of PLMS in English literature. Click here for file

## References

[B1] Baylor SM Berg JW Cross-classification and survival characteristics of 5,000 cases of cancer of the pancreas J Surg Oncol 1973 5 335 358 4355621 10.1002/jso.2930050410

[B2] ROSS CF Leiomyosarcoma of the pancreas Br J Surg 1951 39 53 56 14858827 10.1002/bjs.18003915311

[B3] Du X Cheng Z Zhou ZG Zhang MM Chen ZX Primary pancreatic leiomyosarcoma: a retrospective analysis of clinical characteristics and prognosis of this rare disease Hepatogastroenterol 2012 59 2644 2649 10.5754/hge12273 22641076

[B4] Feinberg SB Margulis AR Lober P Roentgen findings in leiomyosarcoma of the pancreas Minn Med 1957 40 505 506 13451475

[B5] Izumi H Okada K Imaizumi T Hirabayashi K Matsuyama M Dowaki S Tobita K Makuuchi H Leiomyosarcoma of the pancreas: report of a case Surg Today 2011 41 1556 1561 21969162 10.1007/s00595-010-4536-1

[B6] Aihara H Kawamura YJ Toyama N Mori Y Konishi F Yamada S A small leiomyosarcoma of the pancreas treated by local excision HPB (Oxford) 2002 4 145 148 18332943 10.1080/136518202760388064 PMC2020541

[B7] Hur YH Kim HH Park EK Seoung JS Kim JW Jeong YY Lee JH Koh YS Kim JC Kim HJ Cho CK Primary leiomyosarcoma of the pancreas J Korean Surg Soc 2011 Suppl 1 S69 S73 22319744 10.4174/jkss.2011.81.Suppl1.S69 PMC3267071

[B8] Srivastava DN Batra A Thulkar S Julka PK Leiomyosarcoma of pancreas: imaging features Indian J Gastroenterol 2000 19 187 188 11059188

[B9] Sato T Asanuma Y Nanjo H Arakawa A Kusano T Koyama K Shindo M A resected case of giant leiomyosarcoma of the pancreas J Gastroenterol 1994 29 223 227 8012514 10.1007/BF02358688

[B10] Nesi G Pantalone D Ragionieri I Amorosi A Primary leiomyosarcoma of the pancreas: a case report and review of literature Arch Pathol Lab Med 2001 125 152 155 11151070 10.5858/2001-125-0152-PLOTP

[B11] Komoda H Nishida T Yumiba T Nishikawa K Kitagawa T Hirota S Ito T Matsuda H Primary leiomyosarcoma of the pancreas–a case report and case review Virchows Arch 2002 440 334 337 11889607 10.1007/s00428-001-0557-x

[B12] Berman JK Levene N Sarcoma of the pancreas AMA Arch Surg 1956 73 894 896 13361757 10.1001/archsurg.1956.01280050162030

[B13] Becker WF Welsh RA Pratt HS Cystadenoma and cystadenocarcinoma of the pancreas Ann Surg 1965 161 845 863 14295937 10.1097/00000658-196506000-00005 PMC1409085

[B14] Ishikawa O Matsui Y Aoki Y Iwanaga T Terasawa T Wada A Leiomyosarcoma of the pancreas. Report of a case and review of the literature Am J Surg Pathol 1981 5 597 602 7325276 10.1097/00000478-198109000-00009

[B15] Lakhoo K Mannell A Pancreatic leiomyosarcoma. A case report S Afr J Surg 1991 29 59 60 1882318

[B16] Russ PD Leiomyosarcoma of the pancreatic bed detected on CT scans AJR Am J Roentgenol 1993 161 210 8517309 10.2214/ajr.161.1.8517309

[B17] de Alava E Torramade J Vazquez JJ Leiomyosarcoma of the pancreas Virchows Arch A Pathol Anat Histopathol 1993 422 419 422 8322457 10.1007/BF01605462

[B18] Ishii H Okada S Okazaki N Nose H Yoshimori M Aoki K Tsuda H Hirohashi S Leiomyosarcoma of the pancreas: report of a case diagnosed by fine needle aspiration biopsy Jpn J Clin Oncol 1994 24 42 45 8126920

[B19] Peskova M Fried M Pancreatic tumor of mesenchymal origin–an unusual surgical finding Hepatogastroenterol 1994 41 201 203 8056415

[B20] Aranha GV Simples PE Veselik K Leiomyosarcoma of the pancreas Int J Pancreatol 1995 17 95 97 8568340 10.1007/BF02788364

[B21] Shimizu M Hirokawa M Matsumoto T Iwamoto S Manabe T Fatty replacement of the pancreatic body and tail associated with leiomyosarcoma of the pancreatic head Pathol Int 1997 47 633 636 9311016 10.1111/j.1440-1827.1997.tb04554.x

[B22] Owen CH Madden JF Clavien PA Spindle cell stromal tumor of the pancreas: treatment by pancreatoduodenectomy Surgery 1997 122 105 111 9225922 10.1016/s0039-6060(97)90271-3

[B23] Chawla S Gairola M Nachiappan PL Deshpande A Rathi AK Rath GK Pancreatic leiomyosarcoma in a middle-aged lady Trop Gastroenterol 1998 19 118 119 9828713

[B24] Paciorek ML Ross GJ MR imaging of primary pancreatic leiomyosarcoma Br J Radiol 1998 71 561 563 9691904 10.1259/bjr.71.845.9691904

[B25] Zalatnai A Kovacs M Flautner L Sipos B Sarkady E Bocsi J Pancreatic leiomyosarcoma. Case report with immunohistochemical and flowcytometric studies Virchows Arch 1998 432 469 472 9645448 10.1007/s004280050193

[B26] Ferlan-Marolt V Vladislav P Alojz P Pancreatic leiomyosarcoma: clinicopathohistological presentation of a rare tumor Hepatogastroenterol 2000 47 556 559 10791237

[B27] Machado MC Cunha JE Penteado S Bacchella T Jukemura J Costa AC Halpern-Salomon I Preoperative diagnosis of pancreatic leiomyosarcoma Int J Pancreatol 2000 28 97 100 11128979 10.1385/IJGC:28:2:097

[B28] Deveaux PG Aranha GV Yong S Leiomyosarcoma of the pancreas HPB (Oxford) 2001 3 175 177 18332921 10.1080/136518201317077206 PMC2020799

[B29] Maarouf A Scoazec JY Berger F Partensky C Cystic leiomyosarcoma of the pancreas successfully treated by splenopancreatectomy: a 20-year follow-up Pancreas 2007 35 95 98 17575551 10.1097/01.mpa.0000278689.86306.70

[B30] Muhammad SU Azam F Zuzana S Primary pancreatic leiomyosarcoma: a case report Cases J 2008 1 280 18957130 10.1186/1757-1626-1-280 PMC2584078

[B31] Riddle ND Quigley BC Browarsky I Bui MM Leiomyosarcoma arising in the pancreatic duct: a case report and review of the current literature Case Rep Med 2010 2010 252364 20589089 10.1155/2010/252364 PMC2892659

[B32] Zhang H Jensen MH Farnell MB Smyrk TC Zhang L Primary leiomyosarcoma of the pancreas: study of 9 cases and review of literature Am J Surg Pathol 2010 34 1849 1856 21107091 10.1097/PAS.0b013e3181f97727

[B33] Zhang QY Shen QY Yan S Zheng SS Primary pancreatic pleomorphic leiomyosarcoma J Int Med Res 2011 39 1555 1562 21986161 10.1177/147323001103900447

[B34] Vanderpuye V Clegg-Lamptey JN Yarney J Aryeetey N Metastatic primary leiomyosarcoma of the pancreas to the liver: report of a surgically treated case J Gastrointest Cancer 2012 43 70 72 21720738 10.1007/s12029-011-9299-4

[B35] Moletta L Sperti C Beltrame V Gruppo M Blandamura S Pasquali C Pedrazzoli S Leiomyosarcoma of the pancreas with liver metastases as a paradigm of multimodality treatment: case report and review of the literature J Gastrointest Cancer 2012 43 246 250 10.1007/s12029-012-9405-2 22733567

[B36] Nordback I Mattila J Tarkka M Resectable leiomyosarcoma of inferior vena cava presenting as carcinoma of the pancreas. Case report Acta Chir Scand 1990 156 577 580 2239058

