# Supplementary material for: Clinical characteristics and prognosis of primary leiomyosarcoma of the pancreas: a systematic review
Source: World J Surg Oncol. 2013 Nov 12;11:290. doi: 10.1186/1477-7819-11-290 (PMC3874640; doi:10.1186/1477-7819-11-290)
Supplement: Additional file 1 — Reported cases of PLMS in Chinese literature. [file 1477-7819-11-290-S1.pdf]

Additional file 1 Reported cases of primary pancreatic leiomyosarcoma in Chinese literature

Case	Author	Year	Age(years old)/ Gender	Clinical manifestation	Size (cm)	Locations	Morpho logy	Metastasis or invasion	Surgery	Follow up
**1**	Zhao	1982	58/F	Mass	10	Head	…	Duodenum	Nonresectable	Died, 3 mon
**2**	Wei	1985	48/M	Mass,abdominal distention,weight loss, anaemia	20	Body-tail	Solid	stomach , spleen,SMA，transverse mesocolon	Nonresectable	…
**3**	Feng	1988	46/F	Mass,abdominal pain	14	Body-tail	Cystic	Stomach , duodenum, transverse mesocolon	Nonresectable	Died, 8 mon
**4**	Shao	1989	65/F	Jaundice,weight loss	8	Head	Mixed	Hepatoduodenal, ligament	PD	
**5**	Chen	1990	41/F	Mass	14	Tail	Solid	No	DP	Alive, 36 mon
**6**	Zhang	1992	52/M	Mass	13	Body	Mixed	Regional invasion	Nonresectable	Died, 4 mon
**7,8**	Wei	1992	49/F	Mass,diarrhea, anaemia, melena,fever	14	Head	Solid	No	PD	Alive, 24 mon
			61/M	Mass,anaemia,melena	20	Head	Solid	No	PD	Alive, 36 mon
**9**	Du	1993	55/M	Mass,gastrointestinal bleeding,cachexia	5	Body	Solid	Stomach	DP+Gastrostomy	spread to multiple organs, died at 8 mon
**10**	Liu	1993	56/F	Abdominal pain, loss of appetite	10	Body-tail	Mixed	No	DP+splenectomy	…
**11**	Zhang	1994	46/F	Mass	16	Tail	Solid	No	Caudal pancreatectomy	Alive, 6 mon
**12**	Dong	1994	41/F	Mass	26	Body-tail	Solid	No	DP+splenectomy	…

**13**	Zhang	1997	53/F	Mass,weight loss, vomiting,anaemia gastrointestinal bleeding	25	Tail	Cystic	No	Cystectomy	spread to multiple organs, died at 16 mon
**14**	Yu	1998	45/M	Mass,weight loss, abdominal distention	18	Head	Mixed	PV,SMA,SMV	Partial resection	…
**15**	Wang	1998	45/M	Mass, abdominal distention	18	Head	…	SMA,SMV, transverse mesocolon	Nonresectable	Died, 6 mon
**16**	Wang	1998	63/M	Mass	14	Body-tail	Cystic	No	DP	Died, 12 mon
**17**	Qian	2001	68/M	Mass,fever, anaemia	30	Tail	Cystic	Stomach, spleen	DP+splenectomy +Gastrostomy	…
**18**	Hua	2005	40/M	Mass, abdominal pain	7.8	Head	Mixed	Widespread metastases	Nonresectable	…
**19**	Tang	2009	51/F	Mass	8	Body-tail	Mixed	No	DP	Alive, 39 mon
**20**	Wang	2012	15/F	Abdominal pain, Jaundice	8	Head	Mixed	No	PD	Alive, 48 mon

… The data are not recorded in the article.

Abbreviations: SMA, superior mesenteric artery; SMV, superior mesenteric vein; PV, portal vein; PD, pancreatoduodenectomy; DP, distal pancreatectomy, mon, month.
