# Supplementary material for: Clinical characteristics and prognosis of primary leiomyosarcoma of the pancreas: a systematic review
Source: World J Surg Oncol. 2013 Nov 12;11:290. doi: 10.1186/1477-7819-11-290 (PMC3874640; doi:10.1186/1477-7819-11-290)
Supplement: Additional file 2 — Reported cases of PLMS in English literature. [file 1477-7819-11-290-S2.doc]

**Additional file 2 Reported cases of primary pancreatic leiomyosarcoma in English literature**

| **Case** | **Author** | **Year** | **Age(years old)/**  **Gender** | **Clinical manifestation** | **Size**（**cm)** | **Locations** | **Morphology** | **Metastasis**  **or invasion** | **Surgery** | **Follow up** |
| --- | --- | --- | --- | --- | --- | --- | --- | --- | --- | --- |
| **1** | Ross[2] | 1951 | 80/M | Mass,weight loss | … | Whole pancreas | Solid | Widespread  metastases | Autopsy | … |
| **2** | Berman[12] | 1956 | 47/M | Jaundice,  weight loss | 5.5 | Head | Mixed | No | PD | Alive,19 mon |
| **3** | Feinberg[4] | 1957 | 14/M | Abdominal pain ,nausea | 11 | Head | Mixed | No | PD | … |
| **4** | Becker[13] | 1965 | … | … | … | … | Cystic | … | … | … |
| **5-9** | Baylor[1] | 1973 | 51(Median),  M(3),F(2) | … | … | Localized(1) | … | Regional spread(1), disseminated(3) | … | … |
| **10** | Ishikawa[14] | 1981 | 44/M | Mass,  abdominal pain | 8 | Head | Mixed | No | PD | died with liver metastases at 48 mon |
| **11** | Lakhoo[15] | 1991 | 68/M | Mass,  abdominal pain,  weight loss | 17 | Body | Solid | No | DP,  gastric resection,  and transverse colectomy | Alive, 24 mon |
| **12** | Russ[16] | 1993 | 67/M | abdominal pain, | 10 | Body-tail | … | Liver,spleen,  lymph nodes | Nonresectable | … |
| **13** | de Alava[18] | 1993 | 71/M | abdominal pain, | 3.6 | Body | Solid | No | Pancreatectomy | … |
|  |  |  |  |  |  |  |  |  |  |  |
|  |  |  |  |  |  |  |  |  |  |  |
|  |  |  |  |  |  |  |  |  |  |  |
|  |  |  |  |  |  |  |  |  |  |  |
|  |  |  |  |  |  |  |  |  |  |  |
|  |  |  |  |  |  |  |  |  |  |  |
|  |  |  |  |  |  |  |  |  |  |  |
|  |  |  |  |  |  |  |  |  |  |  |
|  |  |  |  |  |  |  |  |  |  |  |
|  |  |  |  |  |  |  |  |  |  |  |
|  |  |  |  |  |  |  |  |  |  |  |
|  |  |  |  |  |  |  |  |  |  |  |
|  |  |  |  |  |  |  |  |  |  |  |
|  |  |  |  |  |  |  |  |  |  |  |
|  |  |  |  |  |  |  |  |  |  |  |
|  |  |  |  |  |  |  |  |  |  |  |
|  |  |  |  |  |  |  |  |  |  |  |
|  |  |  |  |  |  |  |  |  |  |  |
|  |  |  |  |  |  |  |  |  |  |  |
|  |  |  |  |  |  |  |  |  |  |  |
|  |  |  |  |  |  |  |  |  |  |  |
|  |  |  |  |  |  |  |  |  |  |  |
|  |  |  |  |  |  |  |  |  |  |  |
|  |  |  |  |  |  |  |  |  |  |  |
|  |  |  |  |  |  |  |  |  |  |  |
|  |  |  |  |  |  |  |  |  |  |  |
|  |  |  |  |  |  |  |  |  |  |  |
|  |  |  |  |  |  |  |  |  |  |  |
|  |  |  |  |  |  |  |  |  |  |  |
|  |  |  |  |  |  |  |  |  |  |  |
|  |  |  |  |  |  |  |  |  |  |  |
|  |  |  |  |  |  |  |  |  |  |  |
|  |  |  |  |  |  |  |  |  |  |  |
|  |  |  |  |  |  |  |  |  |  |  |
|  |  |  |  |  |  |  |  |  |  |  |
|  |  |  |  |  |  |  |  |  |  |  |
|  |  |  |  |  |  |  |  |  |  |  |
|  |  |  |  |  |  |  |  |  |  |  |
|  |  |  |  |  |  |  |  |  |  |  |
|  |  |  |  |  |  |  |  |  |  |  |

|  |  |  |  | weight loss |  |  |  |  |  |  |
| --- | --- | --- | --- | --- | --- | --- | --- | --- | --- | --- |
| **14** | Ishii[18] | 1994 | 66/M | Incidental finding | 14 | Tail | Solid | Liver | Nonresectable | spread to multiple organs, died at 33 mon |
| **15** | Sato[9] | 1994 | 53/F | Mass | 25 | Body | Solid | No | DP+splenectomy | … |
| **16** | Peskova[19] | 1994 | 68/F | Melena | 15 | Head | Solid | No | PD | Alive, 36 mon |
| **17** | Aranha[20] | 1995 | 45/F | acute pancreatitis | 3 | Body | Cystic | No | DP | Died, 9 mon |
| **18** | Shimizu[21] | 1997 | 49/F | Abdominal pain,jaundice | 15 | Head | Solid | Widespread metastases | Nonresectable | Died, 3 mon |
| **19** | Owen[22] | 1997 | 40/M | Abdominal pain | 6 | Head | Solid | No | PD | Alive, 120 mon |
| **20** | Chawla[23] | 1998 | 45/F | Mass,  haemoptysis | 9.2 | Head | Solid | Liver,Lung | Nonresectable | Alive, 19 mon |
| **21** | Paciorek[24] | 1998 | 63/F | Abdominal pain,nausea,  vomiting | 3 | body | Solid | No | DP+splenectomy,cholecystectomy, | Alive, 12 mon |
| **22** | Zalatnai[25] | 1998 | 57/M | Abdominal pain, vomiting | 6 | Head | Solid | Duodenum,inferior vena cava | retrocolic  gastroenteroanastomosis(biopsy) | spread to multiple organs, died at 6 mon |
| **23** | Ferlan-Marolt[26] | 2000 | 57/F | Abdominal pain | 12 | Body | Solid | No | DP | Died, 5 days |
| **24-27** | Srivastava  [8] | 2000 | 49/M | Mass,vomiting,  weight loss | … | Body-tail | Cystic | No | DP | … |
|  |  |  | 38/M | Abdominal pain,vomiting,  weight loss | … | Body-tail | … | Regional invasion,peritoneal nodules | Nonresectable | Died, 3 mon |
|  |  |  | 45/M | Mass, | … | Head | … | lung metastasis | Nonresectable | … |
|  |  |  |  |  |  |  |  |  |  |  |
|  |  |  |  |  |  |  |  |  |  |  |
|  |  |  |  |  |  |  |  |  |  |  |
|  |  |  |  |  |  |  |  |  |  |  |
|  |  |  |  |  |  |  |  |  |  |  |
|  |  |  |  |  |  |  |  |  |  |  |
|  |  |  |  |  |  |  |  |  |  |  |
|  |  |  |  |  |  |  |  |  |  |  |
|  |  |  |  |  |  |  |  |  |  |  |
|  |  |  |  |  |  |  |  |  |  |  |
|  |  |  |  |  |  |  |  |  |  |  |
|  |  |  |  |  |  |  |  |  |  |  |
|  |  |  |  |  |  |  |  |  |  |  |
|  |  |  |  |  |  |  |  |  |  |  |
|  |  |  |  |  |  |  |  |  |  |  |
|  |  |  |  |  |  |  |  |  |  |  |
|  |  |  |  |  |  |  |  |  |  |  |
|  |  |  |  |  |  |  |  |  |  |  |
|  |  |  |  |  |  |  |  |  |  |  |
|  |  |  |  |  |  |  |  |  |  |  |
|  |  |  |  |  |  |  |  |  |  |  |
|  |  |  |  |  |  |  |  |  |  |  |
|  |  |  |  |  |  |  |  |  |  |  |
|  |  |  |  |  |  |  |  |  |  |  |
|  |  |  |  |  |  |  |  |  |  |  |
|  |  |  |  |  |  |  |  |  |  |  |

|  |  |  |  | hemoptysis,weight loss,anorexia |  |  |  |  |  |  |
| --- | --- | --- | --- | --- | --- | --- | --- | --- | --- | --- |
|  |  |  | 41/M | Mass | 3.5 | Head | … | No | PD | died with liver metastases at 6 mon |
| **28** | Machado[27] | 2000 | 52/M | Incidentally found | 7.5 | Head | Solid | No | PPPD | Alive, 24 mon |
| **29** | Nesi[10] | 2001 | 76/M | Fever | 8 | Tail | Solid | No | DP+splenectomy | Recurrence,9  mon;died,12 mon |
| **30** | Deveaux[28] | 2001 | 44/F | Mass, hypoglycemia | 5 | Head | Mixed | Duodenal wall | PD | Alive, 48 mon |
| **31** | Komoda[11] | 2002 | 52/F | Jaundice | 1.5 | Head | Solid | Duodenal wall | PPPD | Alive,12 mon |
| **32** | Aihara[6] | 2002 | 25/F | back pain | 3.5 | Body | Cystic | No | Local excision | Alive. 42 mon |
| **33** | Maarouf[29] | 2007 | 40/F | Abdominal pain | 5 | Tail | Cystic | No | DP+splenectomy | Alive, 240 mon |
| **34** | Muhammad  [30] | 2008 | 73/M | abdominal pain,  weight loss,  anorexia,  jaundice | 10 | Body | Mixed | Liver | Nonresectable | Died, 3 mon |
| **35** | Riddle[31] | 2010 | 83/F | Abdominal pain , weight loss | 8.2 | Tail | Mixed | No | DP+splenectomy | Alive, 8 mon |
| **36-44** | Zhang[32] | 2010 | 69/F | Incidentally detected | NA | Tail | … | Liver | Biopsy | Died, 13 mon |
|  |  |  | 78/M | Incidentally detected | 30 | Head | … | No | Biopsy | Died of HCC 12 mon |
|  |  |  | 69/M | Jaundice, | NA | Head | … | Liver | Palliative | Died, 27 mon |
|  |  |  |  |  |  |  |  |  |  |  |
|  |  |  |  |  |  |  |  |  |  |  |
|  |  |  |  |  |  |  |  |  |  |  |
|  |  |  |  |  |  |  |  |  |  |  |
|  |  |  |  |  |  |  |  |  |  |  |
|  |  |  |  |  |  |  |  |  |  |  |
|  |  |  |  |  |  |  |  |  |  |  |
|  |  |  |  |  |  |  |  |  |  |  |
|  |  |  |  |  |  |  |  |  |  |  |
|  |  |  |  |  |  |  |  |  |  |  |
|  |  |  |  |  |  |  |  |  |  |  |
|  |  |  |  |  |  |  |  |  |  |  |
|  |  |  |  |  |  |  |  |  |  |  |

|  |  |  |  | abdominal pain |  |  |  |  | procedure |  |
| --- | --- | --- | --- | --- | --- | --- | --- | --- | --- | --- |
|  |  |  | 65/F | Abdominal pain,  weight loss | 8 | Head | … | Liver | Palliative procedure | Died, 5 mon |
|  |  |  | 87/M | Abdominal pain,  weight loss | 6 | Tail | … | No | Palliative procedure | Alive, 10 mon |
|  |  |  | 56/F | Abdominal pain | 12.5 | Head | … | Liver | PD | Died, 12 mon |
|  |  |  | 41/M | Abdominal pain,  weight loss | 1 | Head | … | No | PD | Alive, 66 mon |
|  |  |  | 63/F | Jaundice, abdominal pain,  weight loss | 11.5 | Head | … | NO | PD | Died, 98 mon |
|  |  |  | 39/M | Jaundice,  weight loss | 5.7 | Head | … | No | PD | Alive, 44 mon |
| **45** | Hur[7] | 2011 | 70/F | Abdominal discomfort | 5 | Head | Solid | No | PPPD+regional lymph node dissection | Liver metasistasis,6 mon;died,22 mon |
| **46** | Izumi[5] | 2011 | 41/F | Found incidentally | 4.5 | Body | Solid | No | DP+splenectomy | Alive, 14 mon |
| **47** | Zhang[33] | 2011 | 56/F | Mass,  abdominal pain | 13 | Body-tail | Solid | No | DP | Alive, 14 mon |
| **48** | Vanderpuye  [34] | 2011 | 59/M | Mass, anorexia  weight loss | 24 | Tail | Mixed | Liver,  transverse colon. | DP with  Resection attached of transverse colon | Alive, 24 mon |
| **49** | Moletta[36]. | 2012 | 54/F | Mass | 13 | Body-tail | Mixed | No | DP, | Alive, 37 mon |
|  |  |  |  |  |  |  |  |  |  |  |

|  |  |  |  |  |  |  |  |  | left hepatectomy |  |
| --- | --- | --- | --- | --- | --- | --- | --- | --- | --- | --- |

… The data are not recorded in the article.

Abbreviations: PD, pancreatoduodenectomy; DP, distal pancreatectomy, PPPD, pylorus-preserving pancreaticoduodenectomy; mon, month.
